# Experimental Study on the Shear Strength of Reinforced Concrete Beams with Various Integrated Shear Reinforcements

**DOI:** 10.3390/ma15093091

**Published:** 2022-04-24

**Authors:** Mooyoung Yoo

**Affiliations:** Division of Smart Architecture and Civil Engineering, Daejin University, Pocheon 11159, Korea; myyoo@daejin.ac.kr

**Keywords:** integrated shear reinforcements, shear strength, reinforced concrete beam

## Abstract

The purpose of this study was to evaluate the shear performance of concrete beams with integrated shear reinforcements made of steel plates and rebar bent in an N shape (N-type rebar), and to evaluate the applicability of the current relevant design standards. For this purpose, four concrete beam specimens were manufactured. Four-point loading tests were performed with all the specimens. The experiments confirmed that both types of shear reinforcements had a shear-reinforcing effect (an about 60% increase in shear strength), but the N-type rebar did not exceed the nominal shear strength, probably because the rebar did not yield sufficiently. A sufficient number of steel-plate-type shear reinforcements yielded in the shear crack. When evaluating the shear performance of a new shear reinforcement, it is necessary to calculate the design strength by actually reflecting whether the shear reinforcements’ yields are due to the angle of the diagonal crack. Calculating the shear contribution based on the strain of the shear reinforcements and comparing this shear strength with those five design standards, the shear strength of the shear reinforcements were evaluated conservatively. It is considered that there will be no problem in structural safety even if the shear design is carried out according to the current design standards.

## 1. Introduction

The shear behavior of reinforced concrete beams is very difficult to predict because various influencing factors such as the member shape, the concrete compressive strength, the strength and details of the shear reinforcement, the tensile reinforcement ratio, and the shear reinforcement ratio have a complex effect [1]. In addition, since the shear failure of reinforced concrete beams is the result of a complex action of various fractures such as diagonal cracks, concrete strut crushing, bond failure between the main rebar and concrete, and shear rebar yielding and fracture, it is difficult to predict their ultimate strength and failure points [2].

Generally, in reinforced concrete beams, shear reinforcing bars such as stirrups are used as shear reinforcements to prevent brittle failure and induce flexural behavior. Shear reinforcement transfers stress between shear cracks to prevent the shear failure of concrete and to give it greater strength and ductility. However, in the case of the existing shear reinforcement details such as stirrups, the construction process is complicated by segmented details. Previous studies have been conducted on various existing segmented shear reinforcements.

Choi et al. [3] evaluated the shear reinforcement performance of fiber-reinforced polymer (FRP) plates. Their testing confirmed that the shear strength of the reinforced concrete beam with the FRP plates was improved, and that the shear reinforcement effect was greater when the shear reinforcement was attached perpendicularly to the crack angle. Lee et al. [4] experimented with shear-reinforced beams with slit-type steel plates attached to the beam surface. Their paper reported that with the vertical slit steel plate retrofit, the shear strength greatly depended on the increase in the thickness of the steel plate and a larger attachment area between the steel plate and the concrete. They also confirmed that the slit-type steel plate with a 45-degree inclination was more effective. Although the maximum strength did not change as the attachment area changed, the shear stiffness changed as the concrete crushing point changed. Sharif et al. [5] studied shear reinforcement by attaching steel plates and U-strips to the surface of reinforced concrete beams, and proposed design equations for this. They reported that the steel plate and the U-strip were effective in enhancing shear strength and could improve ductility. F. Qin et al. [6] conducted an experimental study on the shear strength of a new shear reinforcement by welding a cross-tie between the upper and lower steel plates. The experimental results were compared with the design shear strength presented by ACI 318 [7] and Modelcode 2010 [8], and a design method was proposed to design the developed shear reinforcement. C. Bywalski et al. [9] conducted an experimental study on the shear strength of reinforced concrete beams reinforced with a glass-fiber-reinforced polymer (GFRP) reinforcement, and compared and evaluated the performance according to GFRP details. K. Pilakoutas et al. [10] used the inclined shear reinforcement as an alternative detail of the shear reinforcement for the flat slab. At this time, the adhesion performance was increased by digging a groove in the shear reinforcement, and the effect of enhancing the shear strength was confirmed.

Thus, the existing research data describe various methods of concrete shear reinforcement and confirm that steel plates have an excellent reinforcement effect, which is further improved when the attachment area is increased. However, in most studies, the “integrated” shear reinforcement was attached to the surface of reinforced concrete beams, and the main purpose was to retrofit. In addition, it is difficult to apply to general reinforced concrete members because there is a limit to its usability, such as applications limited only to composite members.

**Figure 1:**
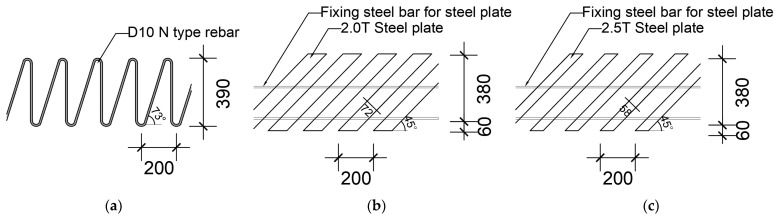
N-type rebar and steel plate shear reinforcement details [unit: mm]. (**a**) N-type rebar. (**b**) steel plate (2.0 T). (**c**) steel plate (2.5 T).

It was necessary to shorten the construction period and improve the workability by omitting the reinforcement process of the existing stirrup at the construction site of the reinforced concrete structure. In addition, it should be usable for general reinforced concrete members. To this end, an integrated shear reinforcement that can be manufactured in a factory has been proposed, and it is necessary to evaluate the structural safety of the new shear reinforcement and to review the applicability of the current design standards. These were manufactured using steel plates and rebar bent in N shapes (N-type rebar) instead of stirrups. The shapes of the N-type rebar and the steel plate shear reinforcements are shown in Figure 1.

To equalize the spacing between the two types of shear reinforcements, the angle of the inclined rebars in the N-type shear reinforcement was about 73°, and the steel plate shear reinforcement was manufactured to have an inclination of 45°. In the steel plate reinforcement, steel wires were welded to the central and lower parts for integration. Previous studies [4,5,11] confirmed that the performance of shear reinforcement made of steel plates varies depending on the attachment area in contact with the concrete. In this study, specimens with the same size but different areas contacting the concrete were fabricated, and the shear performance of the contact areas was evaluated together. Four-point loading tests of the reinforced concrete beams were conducted, and the shear strength of this newly developed shear reinforcement was compared with five current design standards (Section 2).

## 2. Current Design Standards

In this study, the current design standards of several countries were analyzed to determine whether the newly developed shear reinforcement can meet their requirements. In ACI 318-19 [7], except for members that can be designed according to the strut-tie model, the design shear strength $\phi V_{n}$ is required to be designed to satisfy the factored shear force $V_{u}$ or more in terms of shear force. Here, the nominal shear strength is defined as the sum of the contributions to the concrete and the shear reinforcement, and the design of the shear reinforcement is based on the modified truss analogy. At this time, the angle of the truss diagonal member is defined as 45°. The design equations presented by these ACI provisions are Equations (1)–(5):

(1) $$\mathrm{Nominal}\;\mathrm{shear}\;\mathrm{strength}:\;V_{n}=V_{c}+V_{s}$$

(2) $$\mathrm{Simplified}\;\mathrm{form}:\;V_{c}=0.17\lambda \sqrt{f_{c}\prime }b_{w}d$$

(3) $$\mathrm{Detailed}\;\mathrm{form}:\;V_{c}=\left(0.16\lambda \sqrt{f_{c}\prime }+17\rho_{w}\frac{V_{u}d}{M_{u}}\right)b_{w}d$$

(4) $$\mathrm{Vertical}\;\mathrm{stirrups}:\;V_{s}=\frac{A_{v}f_{yt}d}{s}$$

(5) $$\mathrm{Inclined}\;\mathrm{stirrups}:\;V_{s}=\frac{A_{v}f_{yt}\left(\sin\alpha +\cos\alpha \right)d}{s}$$

Here, $V_{n}$: nominal shear strength, $V_{c}$: shear contribution of concrete, $V_{s}$: shear contribution of stirrup, $f_{c}^{\prime }$: concrete compressive strength, λ: coefficient of lightweight concrete, $b_{w}$: width of beam, d: effective depth of beam, $\rho_{w}$: longitudinal reinforcement ratio, $V_{u},\;M_{u}$: factored shear force and moment, $A_{v}$: Area of shear reinforcement, $f_{yt}$: yielding stress of shear reinforcement, s: spacing of shear reinforcement, α: angle of shear reinforcement.

The shear design of Eurocode2 (EC2) [12] corresponds to the variable angle truss model, and an indirect method is applied to increase the contribution component of the shear reinforcement by reducing the inclination angle without directly reflecting the shear component contributed by concrete. In the shear design of EC2, the three types of design shear strength $V_{d}$ are: the shear strength of members that do not require shear reinforcement ($V_{cd}$), the shear strength of members requiring shear reinforcement ($V_{sd}$), and the maximum design shear strength based on the crushing of the web concrete diagonal compression field. The design equations presented in EC2 are Equations (6) and (7):

(6) $$\begin{array}{c} \mathrm{Designed}\;\mathrm{shear}\;\mathrm{resistance}\;\mathrm{of}\;\mathrm{member}\;\mathrm{without}\;\mathrm{stirrups}:\;\\ V_{cd}=\left[0.18\phi_{c}\left(1+\sqrt{\frac{200}{d}}\right){\left(\rho_{w}f_{ck}\right)}^{\frac{1}{3}}+0.15f_{cp}\right]b_{w}d \end{array}$$

(7) $$\begin{array}{c} \mathrm{Designed}\;\mathrm{shear}\;\mathrm{resistance}\;\mathrm{of}\;\mathrm{member}\;\mathrm{with}\;\mathrm{stirrups}:\;\\ V_{sd}=\frac{\phi_{s}f_{yt}A_{v}z}{s}\cot\theta \leq V_{d,max},\;V_{d,max}=\frac{\phi_{c}vf_{ck}b_{w}z}{\cot\theta +\tan\theta } \end{array}$$

Here, $\phi_{c}$: partial safety factor (concrete), $f_{cp}$: axial stress applying on the section, $\phi_{s}$: partial safety factor (steel), z: internal lever arm, θ: angle of inclination of diagonal compressive stress.

In the fib Model Code 2010 (MC 2010) [8], the shear strength ($V_{Rd}$) of reinforced concrete members is calculated as the sum of the shear strength contributed by the concrete ($V_{Rd,c}$) and the shear strength contributed by the shear reinforcement ($V_{Rd,s}$). There are three shear design levels that consider whether or not to include the shear strength due to the concrete. In the shear design Levels 1 and 2, the shear strength contribution by the concrete ($V_{Rd,c}$) is ignored, and only the shear strength contribution by the shear reinforcement ($V_{Rd,s}$) is considered. On the other hand, in the shear design Level 3, the shear strength contribution by the shear reinforcement ($V_{Rd,s}$) as well as the shear strength contribution by the concrete ($V_{Rd,c}$) are taken into account. The design equations presented in MC 2010 are Equations (8)–(11):

(8) $$V_{Rd}=V_{Rd,c}+V_{Rd,s}\geq V_{Ed},\;V_{Rd}\leq V_{Rd,max}$$

(9) $$V_{Rd,c}=k_{v}\frac{\sqrt{f_{c}^{\prime }}}{\gamma_{c}}zb_{w}$$

(10) $$V_{Rd,s}=\frac{A_{v}}{s}zf_{yt}\cot\theta$$

(11) $$V_{Rd,max\;}=k_{c}\frac{f_{c}^{\prime }}{\gamma_{c}}b_{w}z\sin\theta \cos\theta$$

Here, $k_{v}$: coefficient determined according to shear design level, $\gamma_{c}$: partial safety factor of concrete, $k_{c}$: design shear resistance, $k_{c}$: strength reduction factor.

In the shear design specifications of the Japan Society of Civil Engineering (JSCE) [13], the design shear strength ($V_{yd}$) is expressed as the sum of the contribution of the concrete ($V_{cd}$) and the contribution of the shear reinforcement ($V_{sd}$). $V_{sd}$ is the shear strength calculated assuming the yield of the shear reinforcement, and it is calculated from the truss theory. The JSCE design equations are Equations (12)–(18):

(12) $$V_{Rd}=V_{cd}+V_{sd}+V_{ped}$$

(13) $$V_{cd}=\beta_{d}\beta_{p}\beta_{n}f_{vcd}b_{w}d/\gamma_{b}$$

(14) $$\beta_{d}=\sqrt[{4}]{1000/d}<1.5$$

(15) $$\beta_{p}=\sqrt[{3}]{100p_{v}}<1.5$$

(16) $$\beta_{n}=1+\frac{2M_{o}}{M_{ud}}\;(N_{d}^{\prime }\geq 0)\;\mathrm{or}\;1+\frac{4M_{o}}{M_{ud}}\;(N_{d}^{\prime }<0),\;0\leq \beta_{n}\leq 2$$

(17) $$f_{vcd}=0.2\sqrt[{3}]{f_{cd}^{\prime }}\leq 0.72$$

(18) $$V_{sd}=\left[\frac{A_{s}f_{yt}\left(\sin\alpha_{s}+\cos\alpha_{s}\right)}{s}\right]z/\gamma_{b}$$

Here, $M_{ud}$: pure flexural capacity without consideration of axial force, $f_{cd}^{\prime }$: concrete compressive strength, $\gamma_{b}$: 1.3 may be used in general.

The shear design standard of AASHTO’s LRFD [14] is based on the modified compression field theory (MCFT) developed by Bentz et al. [15]. The nominal shear strength ($V_{n}$) is calculated from that theory as the sum of the shear strength contributed by the concrete ($V_{c}$) and the shear strength contributed by the shear reinforcements ($V_{s}$). The design equations presented in AASHTO’s LRFD are Equations (19)–(22):

(19) $$V_{n}=\min\left(V_{c}+V_{s}+V_{p},\;0.25f_{c}^{\prime }b_{v}d_{v}\right)$$

(20) $$V_{c}=0.083\beta \sqrt{f_{ck}}b_{v}d_{v}$$

(21) $$\mathrm{Vertical}\;\mathrm{stirrups}:\;V_{s}=\frac{\phi_{s}A_{v}f_{yt}d\cot\theta }{s}$$

(22) $$\mathrm{Inclined}\;\mathrm{stirrups}:\;V_{s}=\frac{\phi_{s}A_{v}f_{yt}d(\cot\theta +\cot\alpha )\sin\alpha }{s}$$

## 3. Experimental Plan

### 3.1. Specimen Details

**Figure 2:**
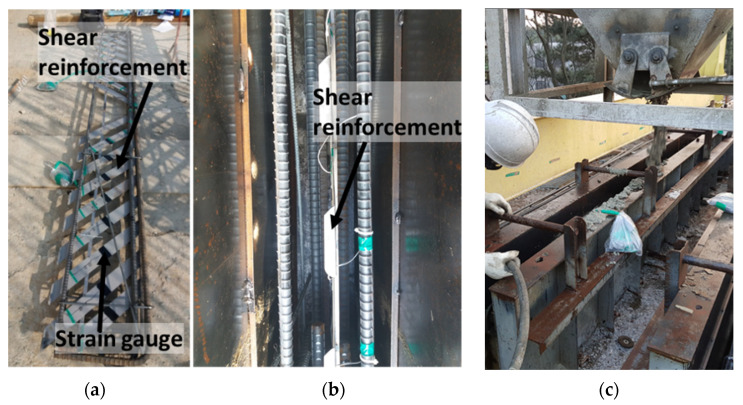
Test specimens’ fabrication. (**a**) reinforcement detail and gauge attachment. (**b**) Formwork installation. (**c**) Concrete casting.

**Figure 3:**
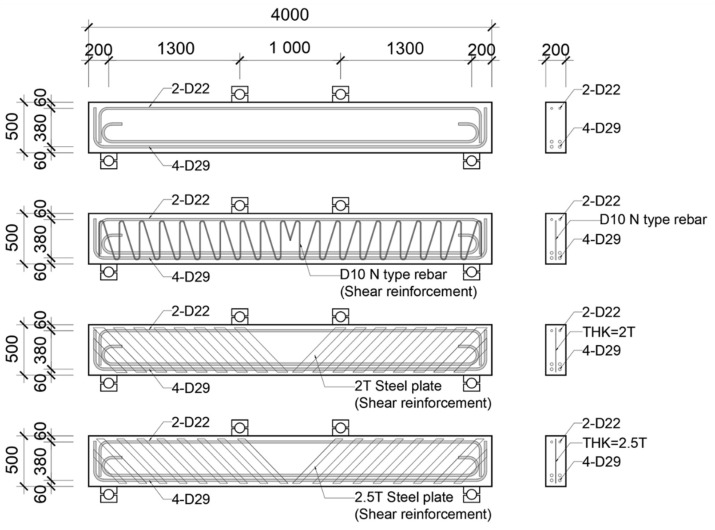
Test specimen details [unit: mm].

To evaluate the performance of the integrated shear reinforcements, all of the specimens were designed so that shear failure could occur before flexural failure. A photograph of the specimen fabrication is shown in Figure 2. The cross-sections and shapes of the specimens are shown in Figure 3, and the experimental parameters and detailed specifications are summarized in Table 1. The main variable was the type of shear reinforcement, which was either non-reinforced, N-type shear reinforcement manufactured by bending D10 rebars, or steel plate reinforcements that were either 2 mm thick × 72 mm wide or 2.5 mm thick × 58 mm wide. The spacing of the shear reinforcements was always identical at 200 mm. In order to prevent the N-type rebar and the steel-plate-type shear reinforcements from tilting to the side, they were bound to the longitudinal rebars using tie wires. The cross-section of the specimens was always set with a width of 200 mm and a height of 500 mm, and four D29 rebars were placed in two layers at the bottom to induce shear failure before flexural failure.

The shear strength calculation assumed that an inclined stirrup was used as a shear reinforcement for the calculation of the design shear strength of the steel plate, as suggested in the current ACI standard 318-19, Equations (23) and (24):

(23) $$\mathrm{Vertical}\;\mathrm{components}\;\mathrm{of}\;\mathrm{shear}\;\mathrm{reinforcement}:\;V_{s}=\frac{A_{v}f_{yt}d}{s}$$

(24) $$\mathrm{Diagonal}\;\mathrm{components}\;\mathrm{of}\;\mathrm{shear}\;\mathrm{reinforcement}:\;V_{s}=\frac{A_{v}f_{yt}\left(\sin\alpha +\cos\alpha \right)d}{s}$$

### 3.2. Test Setup

**Figure 4:**
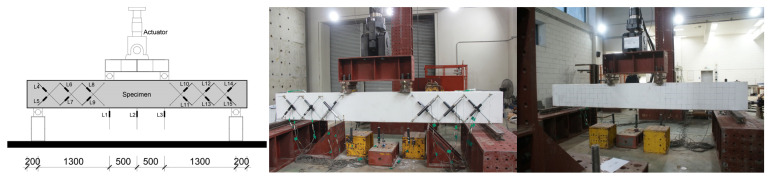
Loading test configuration [unit: mm].

In order to evaluate the integrated shear reinforcement performance, four-point loading tests were performed on the beam specimens, as shown in Figure 4. A simple support setup was created by installing a hinge at 200 mm from the end of the beam specimens, and the position of the loading point was set so that the shear span ratio was 2.95 to induce shear failure. To measure the deflection of the specimens, linear variable differential transformers (LVDTs, Tokyo Measuring Instruments Lab, Tokyo, Japan) were installed at the loading point and the center of the specimen, and also diagonally between the loading point and the supports, to measure the point of the initial shear cracking of the specimens. In addition, a strain gauge was attached to the shear reinforcements for the yield point. The load was applied by displacement control using a 1000 kN actuator, and the load was applied at 0.03 mm per sec. The experiment was terminated when it fell below 80% of the maximum strength.

### 3.3. Material Properties

The specified concrete compressive strength was set to 40 MPa, and the concrete mix proportions are shown in Table 2. The cement is type-1 ordinary Portland cement, and the maximum size of the coarse aggregate is 25 mm, and air-entraining and high-range water-reducing agents are used for admixture. To evaluate the compressive strength of the concrete, three *ϕ* 100 mm × 200 mm specimens were manufactured according to the Korean Agency for Technology and Standards KS F 2403 [16], and the compressive strength of the concrete was evaluated by KS F 2405 [17]. The evaluations of the compressive strength of the specimens were carried out immediately after each beam test. As a result of the concrete material test, the average concrete compressive strength was 49.05 MPa, which exceeded the specified design compressive strength.

The nominal yield strength of the longitudinal reinforcing bar used in the specimens was 500 MPa (SD500), and the upper rebar was D22 and the lower rebar was D29 to induce shear failure. N-type shear reinforcement was manufactured using D10 rebar with a nominal strength of 400 MPa (SD400). Tensile test specimens of the rebar were prepared according to KS B 0801 [18], and the tensile tests were performed according to KS B 0802 [19]. It was confirmed that all the rebars exceeded the nominal yield strength. For the steel plate used to manufacture the steel-plate-type shear reinforcement, SS235 steel with a thickness of either 2 mm or 2.5 mm was used. No. 13 test pieces were manufactured according to KS B 0801. As a result of the material test, the yield strength of the steel plate was similar to the nominal yield strength of 235 MPa. The rebar and steel plate test results are shown in Table 3.

## 4. Experimental Results

### 4.1. Material Properties

**Figure 5:**
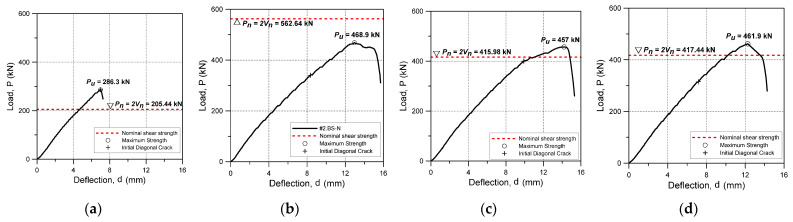
Load-deflection relationships. (**a**) BS-0. (**b**) BS-N. (**c**) BS-2T. (**d**) BS-2.5T.

The experimental results of the reinforced concrete beams with and without shear reinforcement are summarized in Table 4, and the load-displacement relationship for each specimen is shown in Figure 5. As a result of analyzing the strain of the longitudinal reinforcing bar, it was confirmed that shear failure occurred before the tensile reinforcing bar yielded in all specimens, and the experiment was terminated. The experimental results for each specimen are as follows.

The BS-0 specimen was not shear-reinforced; it was designed to evaluate only the shear strength of the concrete and the longitudinal reinforcing bars. As a result of the test, the BS-0 specimen exhibited a typical shear fracture behavior in which the first shear crack occurred at 286 kN, the crack expanded, and the strength rapidly decreased immediately after the shear crack occurred. The actual shear strength of the BS-0 specimen was about 39% higher than the value calculated by applying the material test results to the nominal shear strength equation of ACI 318-19.

The BS-N specimen was designed to evaluate the performance of the N-type rebar shear reinforcement. In this specimen, the initial shear cracking occurred at 340 kN, which was higher than that of the unreinforced BS-0 specimen. Its strength steadily increased after cracking, and then the crack expanded at 468.9 kN and the strength decreased, and finally there was shear fracture. The maximum shear strength of the BS-N specimen was about 17% lower than the nominal shear strength.

The BS-2T specimen was to confirm the shear performance when a 2-mm-thick steel plate shear reinforcement was installed. The BS-2T specimen showed the highest initial shear crack strength among all the specimens, as the initial shear cracking occurred at about 400 kN. After shear cracking, the strength of this specimen continued to rise like the BS-N specimen, but the crack expanded and fractured at 457 kN, which was about 10% higher than the nominal shear strength.

The BS-2.5T specimen was to confirm the shear performance when a 2.5-mm-thick steel plate was installed and the surface area of the shear reinforcement was changed. In this specimen, the first shear cracking occurred at 314.91 kN, and fracture occurred at the maximum strength of 461.9 kN. This was about 11% higher than the nominal shear strength but was not significantly different in strength than the 2T specimen.

### 4.2. Crack and Failure Patterns

**Figure 6:**
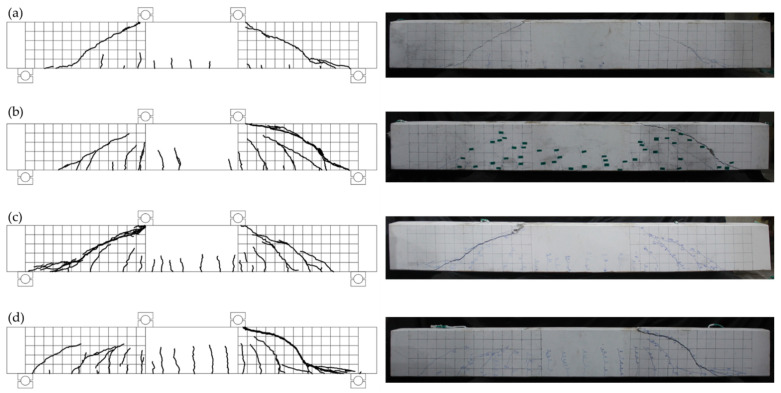
Crack patterns at failure (100-mm unit grid): (**a**) BS-0; (**b**) BS-N; (**c**) BS-2T; (**d**) BS-2.5T.

**Figure 7:**
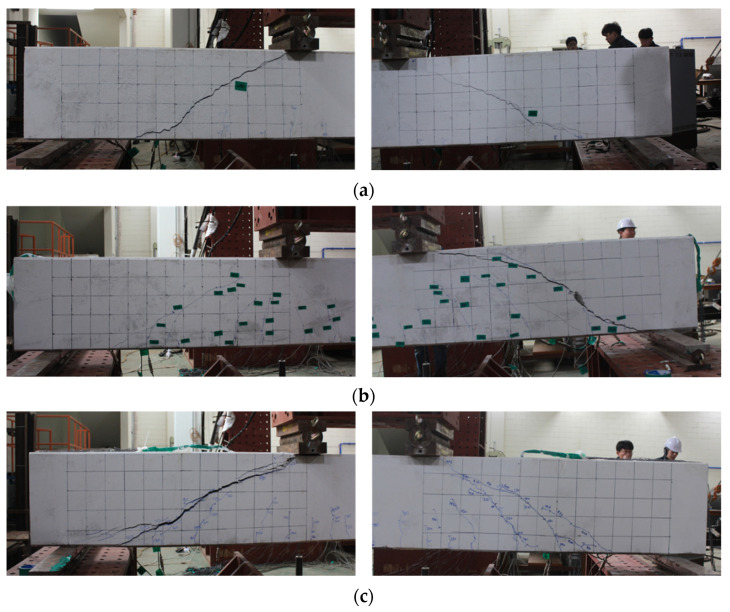

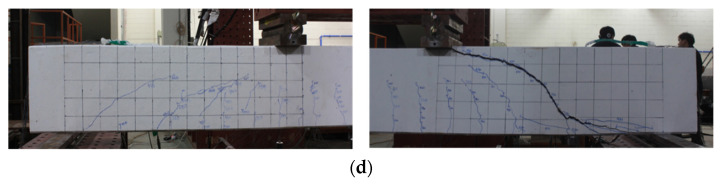
Crack patterns at failure (enlargement). (**a**) BS-0. (**b**) BS-N. (**c**) BS-2T. (**d**) BS-2.5T.

In order to analyze the changes in cracking and fracture patterns according to the types of shear reinforcement, the crack patterns at the final fracture of each specimen are shown with grids in Figure 6 and Figure 7. These cracks were obtained by checking in real time during the experiment, and the crack patterns are shown at the end of the experiment. Here, the size of one grid is 100 mm × 100 mm.

In the BS-0 specimen, which was non-reinforced, after flexural cracking occurred in the lower center of the beam at the initial loading stage, an initial shear crack occurred near the center of the web of the specimen, separately from the flexural cracking. After the initial shear cracks appeared in the specimen web, as the load increased, the shear cracks developed into an arch shape that diagonally connected the support points and the loading points.

In the BS-N specimen with N-type rebar reinforcements, after the initial flexural cracking, flexural cracks progressed diagonally and arcuate shear cracks occurred in the web. Arch-shaped cracks occurred in the form of connecting the support points and the loading points, and it was confirmed that more flexural cracks developed according to the load increase than in the non-reinforced specimen.

The BS-2T specimen reinforced with 2T steel plates also developed an arch-shaped shear crack. A shear splitting crack was confirmed on the support side of the specimen. As the loading progressed, a number of shear cracks eventually merged into an arch-shaped shear crack, and the specimen was fractured.

The BS-2.5T specimen reinforced with 2.5T steel showed diagonal cracks and arcuate cracks in the web after the initial flexural cracks occurred. As the loading progressed, an arcuate crack opened and it was confirmed that the specimen was fractured.

## 5. Evaluation of Shear Strength

### 5.1. Strain of Shear Reinforcements

**Figure 8:**
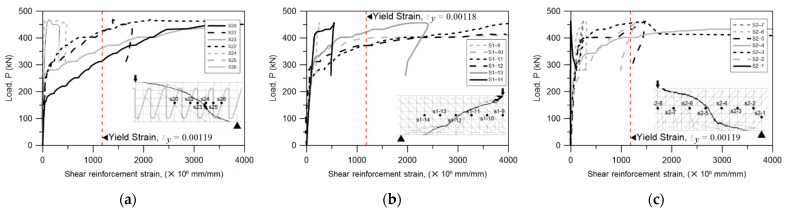
Strain of shear reinforcement. S*x* = strain gauge number. (**a**) BS-N. (**b**) BS-2T. (**c**) BS-2.5T.

The yield strengths of the two types of shear reinforcements studied here were different, so the shear strain according to the load was analyzed in order to determine the yield point and the shear strength of each shear reinforcement in detail. The shear strain was analyzed for the shear reinforcements passing through the crack surface, and six N-type (rebar) shear stiffeners and six steel-plate-type shear stiffeners were analyzed. In the case of the N-type bars, some of the gauges passing through the crack surface did not measure properly, so they were not included in the graph. The yield strains calculated based on the material test results were used, and the results are shown in Figure 8.

In the specimens reinforced with N-type rebars, it was confirmed that most of the strains of the reinforcing bars passing through the crack surface changed rapidly after about 250 kN, but only two of the six rebars yielded at 400 kN. It is possible that the N-type reinforcing bars slipped, because the yield strain did not increase sufficiently even though the strain increased as the rebar was subjected to the shear after shear cracking. Accordingly, the specimen reinforced with the N-type rebar shear reinforcement was destroyed when the nominal shear strength was lower than that of the specimen.

On the other hand, in the specimens reinforced with steel plates, the yield strain was less than that of the N-type rebar shear reinforcement. For this reason, the time-to-yield of the steel plates was earlier than that of the N-type rebar, and four of the six steel plate reinforcements passing through the crack surface increased their strain from about 300 kN and yielded at 350 kN. Accordingly, slip did not occur with the steel plate shear reinforcements, making them more advantageous for post-construction settlement.

### 5.2. Shear Strength of Shear Reinforcements

As mentioned above, ACI 318-19 calculates the design shear strength as the sum of the shear contributions of the concrete and the shear reinforcement using the 45° truss model based on empirical results. Accordingly, in this study, in order to check the shear strength ($V_{\Delta }$) as increased by the shear reinforcement, the shear contribution of the concrete ($V_{c,test}$) was excluded from the experimental results ($V_{u,test}$), as shown in the Equation (25) calculation. Here, the shear contribution of the concrete ($V_{c,test}$) was set as the shear strength of the unreinforced specimen BS-0.

(25) $$V_{\Delta }=V_{u,test}-V_{c,test}$$

Figure 8 shows a comparison between the shear strength increased by the shear reinforcement as calculated by Equation (25) and the experimental results. In the BS-N specimen with N-type rebar shear reinforcement, the strength was 91.31 kN higher than that of the BS-0 specimen, which was the non-reinforced specimen. In the BS-2T specimen reinforced with 2-mm-thick steel plates, its 85.35-kN strength was 60% higher than that of the non-reinforced specimen and 10% higher than the design value.

**Figure 9:**
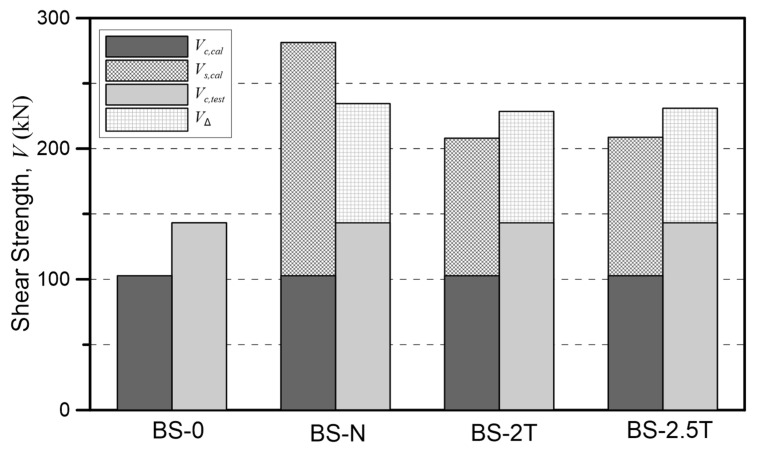
Comparison of shear contributions of the tested reinforcements, rebar, and steel plates.

Thus, the shear strength increased by the shear reinforcement can be calculated through Equation (25), but it is difficult to quantitatively determine the shear contributions of the different shear reinforcement types. The reason is that the shear contribution of the concrete at the point of maximum strength decreases somewhat due to the interaction between the reinforcing bars and the concrete when such reinforcement is added. As can be seen in Figure 9, the shear strength of the rebar and steel plate specimens increased compared to the specimen without reinforcement, but the shear contribution by the concrete itself was smaller than the shear strength of the specimen without reinforcement, so it might be judged that $V_{\Delta }$ is the shear contribution of only the shear reinforcement. However, in reality it will be higher than this. Thus, the shear contributions of the rebar and the steel plate shear reinforcements were quantitatively analyzed through the strain of the shear reinforcement.

**Figure 10:**
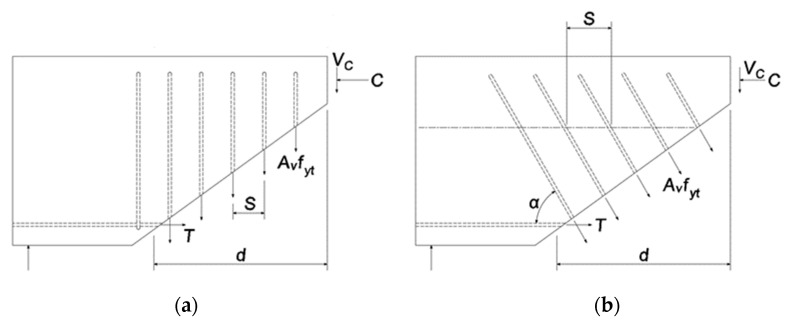
Shear resistance with stirrups. (**a**) Vertical stirrups. (**b**) Inclined stirrups.

In ACI 318-19, the shear contribution of the shear reinforcement is calculated through a free-body diagram of a shear-fractured beam, as shown in Figure 10. When the shear reinforcement is vertically placed, as shown in Figure 10a, and if the reinforcement spacing is *s*, the number of reinforcements across the crack surface is *d*/*s*. At this time, it is assumed that all the shear reinforcements to be cut have yielded.

In contrast, as shown in Figure 10b, when the shear reinforcement is inclined at an angle α and the spacing is *s*, the number *n* of reinforcements crossing the crack surface can be calculated using Equation (26) as follows:

(26) $$n=\frac{d\left(\cot45+\cot\alpha \right)}{s}=\frac{d\left(1+\cot\alpha \right)}{s}$$

Using Equation (26) to calculate the number of shear reinforcements made of N-type rebars and steel plates passing through the crack surface, that is, the number of shear reinforcements that must yield *n* = 6.6 for N-type rebar shear reinforcements and *n* = 4.4 for steel plate reinforcements.

**Figure 11:**
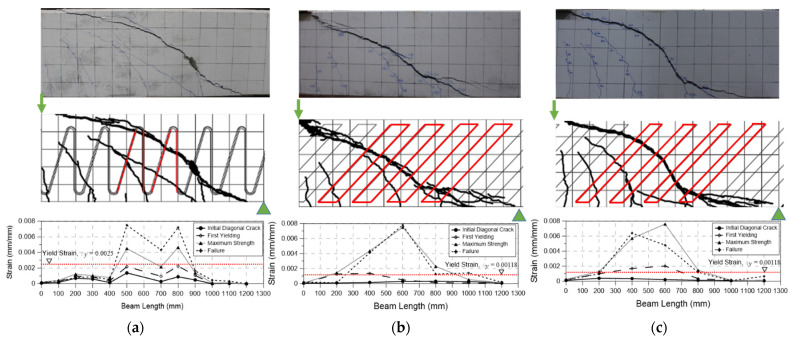
Strain distributions of the rebar and steel plate shear reinforcements. (**a**) BS-N. (**b**) BS-2T. (**c**) BS-2.5T.

In order to determine how many of the rebars and steel plates yielded, the strain of each shear reinforcement was checked; the strain gauge was attached to the middle of each shear reinforcement because the location of the shear crack could not be accurately determined. Because the gauge was located far from the cracking surface, the actual strain of the reinforcement at the cracking surface might have been greater than the measured value due to the adhesion between the concrete and the shear reinforcement, but this effect was neglected to evaluate the experimental results conservatively. Whether or not the shear reinforcement yielded was evaluated based on the strain shown through the material test results of the reinforcing bars and steel plates, the number of shear reinforcements that yielded when the beam reached its maximum strength was measured. The results are shown in Figure 11.

In the case of N-type shear rebar, as shown in Figure 11a, a total of four rebars yielded (shown in red) when the maximum strength was reached, which was less than the number of rebars that had to yield along the crack surface, which was 6.6. As described above, the reason for this result might have been slippage that occurred because the N-type rebar did not fix properly to the concrete because there was no special detail for this. In contrast, because the existing stirrup shear reinforcement surrounded the upper and lower tensile reinforcing bars, it was sufficiently fixed until the yield strength was reached. In the specimens with steel-plate-type shear reinforcements installed (Figure 11b,c), four of the five shear reinforcements passing through the crack surface yielded. This was probably because the shear reinforcement of the steel plates was improved by welding the steel attachment wire, as shown in Figure 1b,c, which unified the steel plate reinforcement.

Considering these experimental results, the contribution to the shear strength of the shear reinforcement ($V_{s,strain}$) was recalculated through the strain of the shear reinforcement passing through the crack surface when the maximum strength was reached, and the results are shown in Table 5. This time, the crack surface was set to the actual crack plane instead of 45° to check the actual shear contribution. Accordingly, the shear contributions of seven N-type rebar shear reinforcements and six steel-plate-type shear reinforcements were evaluated. The number of shear reinforcements that yielded was assumed to be two vertical and two inclined. The yielding of four steel plates was calculated and evaluated, and the results are shown in Table 5.

With the N-type rebar, their shear strength contribution calculated through the strain rate of the shear reinforcement ($V_{s,strain}$) was about 7% smaller than that calculated through the current standards ($V_{s,cal}$). The BS-2T and BS-2.5T strengths were about 1.03 times and 1.01 times larger, respectively, with steel-plate-type shear reinforcements installed. If this is converted into the number of steel plates that yielded, it is about 4.5, which is larger than the 4.4 calculated through Equation (26).

### 5.3. Evaluation of Shear Strength

In order to verify the shear strength according to the design methodology of the integrated shear reinforcement, the experimental results were compared with the nominal shear strength calculated based on the design criteria presented in five reference standards (Table 6). The material strength used in the calculations was based on the material test results. In EC2, the shear strength of the beam is evaluated through the contribution of the shear reinforcement, and this is taken into account. In CEB-FIP Model Code 2010, the shear strength of the specimen is determined according to the level of the shear design, and in this case, the shear strength was calculated as Level 3.

The angle of the concrete strut was calculated as the angle of the sinusoidal crack shown through the experimental results. It was confirmed that the experimental values of the 2T and 2.5T steels in this study were higher than the design values in all of the design standards. This confirms that with integrally manufactured steel shear reinforcement, the shear performance can be improved, and there will be no difficulty in designing using the recommendations in the current design standards.

However, this study focused on the reinforcing effect of the replaceable shear reinforcement for the stirrup with non-seismic detail, and the performance of the member subjected to repeated loads such as seismic load has not been verified yet. Therefore, the integral shear reinforcement that is the subject of this study is limitedly applicable to one-way PC slabs with ribs or simply supported beams, which are structural members that do not resist seismic loads. In future research, it is necessary to develop an integrated shear reinforcement that can resist seismic loads.

## 6. Conclusions

The main purpose of this study is to improve constructability by facilitating the installation of shear reinforcements through the development of new shear reinforcements, and to evaluate structural safety by examining the applicability of current design standards. In this study, experiments were performed on four concrete beam specimens to evaluate the shear performance of integrated shear reinforcement (N-type rebar and steel plates), and the following conclusions were obtained by analyzing the experimental results.

(1) The concrete beam specimen reinforced with N-type rebar showed 182.6-kN higher strength than the non-reinforced specimen, and was 17% lower than the nominal shear strength. It is judged that its strength was lower than the nominal strength because the N-type rebar was not properly attached to the concrete during construction. However, unlike the non-reinforced specimen, with the rebar specimen the fracture occurred with a larger load and greater displacement than in the non-reinforced specimen, as cracks progressed after the initial shear cracking.
(2) The specimens with steel plate shear reinforcement showed higher strength than the non-reinforced specimen, and were about 10% higher than the nominal strength. The fracture occurred as the crack progressed after the initial shear cracking, similar to the N-type rebar shear reinforcement detail. Accordingly, it is judged that the shear strength of the steel-plate-type shear reinforcement can be improved.
(3) In examining the strain rate and stress distribution of each shear reinforcement method to check their shear reinforcement performance, it was confirmed that the N-type rebars were not properly anchored, resulting in slips. Therefore, with N-type rebar shear reinforcement it is necessary to develop appropriate anchoring details.
(4) Calculating the shear contribution in the specimens with steel-plate-type shear reinforcements installed, it was confirmed that it was higher than the current standards. Accordingly, it is judged that there will be no difficulty in designing the newly developed shear reinforcement according to the current standards. However, studies on the surface area and fixation details that can affect the shear performance are still lacking. Therefore, in future studies, there should be detailed consideration of other factors affecting the shear performance of steel-plate-type shear reinforcement. In addition, it is necessary to minimize the uncertainty of shear design through statistical analysis with accumulated data.
(5) In this study, performance evaluation was performed under monotonic loads to evaluate the shear strength improvement effect of shear reinforcements. Therefore, it can be applied only to slabs or simple support beams that do not resist seismic loads. In future studies, it is necessary to study shear reinforcements applicable to members subjected to seismic loads.

## Figures and Tables

**Table 1:** Dimensions and material properties of the specimens.

Specimen	$f_{c}^{\prime }$ (MPa)	$f_{\mathrm{yl}}$ (MPa)	*b* × *h* × *L* (mm)	Type of Shear Reinforcement	Form of Shear Reinforcement	$f_{\mathrm{yt}}$ (MPa)	$\rho_{l}$	*a/d*	*s* (mm)
BS-0	40	500	200× 500× 4000	-	-	-	0.026	2.95	-
BS-N				N-type rebar	D10 rebar	400			200
BS-2T				Steel plate	2 × 72 mm (*t* × *w*)	235			
BS-2.5T					2.5 × 58 mm (*t* × *w*)				

$f_{c}^{\prime }$ is the specified concrete compressive strength, $f_{\mathrm{yl}}$ is yield strength of longitudinal reinforcements, *b* is beam width, *h* is beam height, *L* is beam length, $f_{\mathrm{yt}}$ is yield strength of shear reinforcements, $\rho_{l}$ is longitudinal reinforcement ratio, *a/d* is shear span ratio, *s* is spacing of shear reinforcements.

**Table 2:** Mix proportions of the concrete.

W/B (%)	S/a (%)	Unit Weight (kg/m^3^)				
		W	C	S	G	SP
36.5	50	168	460	883	897	3.6

W/B is water binder ratio, S/a is fine aggregate ratio, W is water, C is cement, S is fine aggregate, G is coarse aggregate, SP is super-plasticizer.

**Table 3:** Rebar and steel plate tensile test results.

Rebar/Steel Plate	$f_{y}$ (MPa)	$f_{u}$ (MPa)	$\varepsilon_{y}$
D10 N-type rebar	503	606	0.0025
D22 upper rebar	508	666	0.0025
D29 lower rebar	552	689	0.0028
2T 2-mm-thick plate	235.5	337.75	0.00118
2.5T 2.5-mm-thick plate	238	332	0.00119

$f_{y}$ is the yield strength of the rebar or steel plate, $f_{u}$ is the ultimate strength of the rebar or steel plate, $\varepsilon_{y}$ is the yield strain of the rebar or steel plate.

**Table 4:** Test results.

Specimen	Calculated Values		Test Results					$\frac{\Delta_{u,test}}{\Delta_{n,cal}}$	$\frac{\Delta_{u,test}}{\Delta_{u,BS-0}}$
	$V_{n,cal}$ (kN)	$\Delta_{n,cal}$ (mm)	$P_{cr,d}$ (kN)	$P_{u,test}$ (kN)	$V_{cr,d}$ (kN)	$V_{u,test}$ (kN)	$\Delta_{u,test}$ (mm)		
BS-0	102.72	5.34	286	286.3	143	143.15	6.98	1.39	-
BS-N	281.32	13.19	340.35	468.9	170.18	234.45	12.96	0.83	1.63
BS-2T	207.99	10.99	400	457	200	228.5	14.2	1.10	1.60
BS-2.5T	208.72	11.1	314.91	461.9	125	230.95	12.26	1.11	1.61

$V_{n,cal}$ is the nominal shear strength calculated by ACI 318-19, $\Delta_{n,cal}$ is the calculated value of deflection at nominal shear strength, $P_{cr,d}$ is the initial diagonal cracking load, $P_{u}$ is the maximum load, $V_{cr,d}$ is the initial diagonal *c* racking strength ($=P_{cr,d}/2$), $V_{u,test}$ is the maximum strength ($=P_{u,test}/2$), and $\Delta_{u,test}$ is the deflection at maximum load.

**Table 5:** Comparison of shear strength.

	$V_{n,cal}$ (kN)	$V_{u,test}$ (kN)	$V_{\Delta }$ (kN)	$\frac{V_{u,test}}{V_{u,cal}}$	$\frac{V_{u,\;test}}{V_{u,BS-0}}$	$V_{s,cal}$ (kN)	$V_{s,strain}$ (kN)	$\frac{V_{s,strain}}{V_{s,cal}}$
BS-0	102.72	143.15	-	1.39	1.00	-		
BS-N	281.32	234.45	91.31	0.83	1.64	178.6	166.2	0.93
BS-2T	207.99	228.5	85.35	1.10	1.6	105.5	108.81	1.03
BS-2.5T	208.72	230.95	87.8	1.11	1.61	107.36	107.48	1.01

$V_{n,cal}$ is the calculated shear strength, $V_{u,test}$ is the maximum shear strength, $V_{\Delta }$ is the shear strength increased by the shear reinforcement, $V_{s,cal}$ is the shear contribution of the shear reinforcement, and $V_{s,strain}$ is the calculated value of the shear contribution of shear reinforcement using shear strain.

**Table 6:** Evaluation of test results through code provisions.

ID	$V_{u}$ (kN)	ACI 318-19		Eurocode2		MC 2010		JSCE		AASHTO LRFD	
		$V_{n}$ (kN)	$V_{u}/V_{n}$	$V_{n}$ (kN)	$V_{u}/V_{n}$	$V_{n}$ (kN)	$V_{u}/V_{n}$	$V_{n}$ (kN)	$V_{u}/V_{n}$	$V_{n}$ (kN)	$V_{u}/V_{n}$
BS-2T	228.5	208.21	1.11	153.35	1.49	161.36	1.42	158.14	1.44	212.56	1.07
BS-2.5T	231.0	210.07	1.10	154.98	1.49	163.47	1.41	158.98	1.45	224.39	1.03

## Data Availability

Data are contained within the article.
